# Serious infections in patients with self-reported psoriatic arthritis from the Psoriasis Longitudinal Assessment and Registry (PSOLAR) treated with biologics

**DOI:** 10.1186/s41927-019-0094-3

**Published:** 2019-11-28

**Authors:** Christopher T. Ritchlin, Mona Stahle, Yves Poulin, Jerry Bagel, Soumya D. Chakravarty, Shelly Kafka, Bhaskar Srivastava, Wayne Langholff, Alice B. Gottlieb

**Affiliations:** 1 Division of Allergy, Immunology, and Rheumatology, University of Rochester Medical Center, 601 Elmwood Ave., Box 695, Rochester, NY 14642 USA; 2 Department of Medicine, Karolinska University Hospital, Karolinska Institutet, Stockholm, Sweden; 3 Université Laval and Centre de Recherche Dermatologique du Quebec métropolitain, Quebec, Canada; 4 Psoriasis Treatment Center of Central New Jersey, East Windsor, NJ USA; 5 Janssen Scientific Affairs, LLC, Horsham, PA USA; 6 Drexel University College of Medicine, Philadelphia, PA USA; 7 Janssen Research & Development, Spring House, PA USA; 8 New York Medical College, Metropolitan Hospital, New York, NY USA

**Keywords:** PSOLAR, Psoriasis, Psoriatic arthritis, Serious infections

## Abstract

**Background:**

Patients with psoriatic arthritis (PsA) have increased risk of adverse events, including serious infections (SI), compared with psoriasis patients.

**Methods:**

Patients eligible for, or receiving conventional systemic and biologic agents for psoriasis were followed prospectively using PSOLAR. Cohorts included: ustekinumab, tumor necrosis factor (TNF) inhibitors; infliximab; etanercept; adalimumab; non-biologic/methotrexate (MTX) (reference group); and non-biologic/non-MTX. Multivariate analyses using Cox hazard regression were used to identify factors associated with time to first SI. Rates of SI in PSOLAR psoriasis patients with self-reported PsA and possible risks with biologic therapy were evaluated.

**Results:**

PSOLAR enrolled 4315 psoriasis patients with self-reported PsA. The overall population (*N* = 2401) included patients (n): 628 ustekinumab; 1413 TNF inhibitors; 258 infliximab; 481 etanercept; 674 adalimumab; 54 other biologics, 98 non-biologic/MTX; 208 non-biologic/non-MTX. Overall, 138 SI were reported with incidence rates per 100 patient-years as follows: a) ustekinumab: 1.00; b) TNF inhibitors: 2.22; c) infliximab: 2.12; d) etanercept: 2.58; e) adalimumab: 1.99; f) non-biologic/MTX: 3.01; g) and non-biologic/non-MTX: 2.31. Age, time-dependent disease activity Physician’s Global Assessment (PGA) of 4, 5, history of infection, and diabetes were associated with increased risk for SI (*p* < 0.05) in self-reported PsA patients. Biologic groups, other than ustekinumab, had numerically higher rates of SI.

**Conclusions:**

PSOLAR psoriasis patients with self-reported PsA in the TNF inhibitors, infliximab, adalimumab, etanercept, and MTX cohorts had numerically higher SI rates than the ustekinumab cohort, although not statistically significant. Age, PGA 4, 5, history of infection, and diabetes were associated with an increased risk for SI, irrespective of biologic exposure.

**Trial registration:**

NCT00508547; Registered July 30, 2007.

## Background

Psoriatic arthritis (PsA) is an immune-mediated inflammatory disease that typically involves inflammation of the peripheral joints, enthesitis, dactylitis, spondylitis, and psoriasis [1]. PsA can be aggressive and therefore result in decreased function in daily activities, impaired health-related quality of life, and increased mortality [1–3]. Due to the chronic inflammatory nature of this disease, patients often require long-term treatment. Several studies have reported increased risk of infections with biologic use in patients with psoriasis [2, 4–7] and rheumatoid arthritis (RA) [2, 8–12]. Likewise, patients with PsA have been reported to have an increased risk of comorbidities and adverse events (AEs), including serious infections (SIs), compared with patients with psoriasis [13, 14]. Many investigators question whether the increased risk for AEs is due to the underlying inflammatory nature of psoriatic disease itself, or to the therapies used to treat the disease.

The impact of biologic use and infection risk is an area of significant interest to the rheumatology community. An unmet need remains for longitudinal data to better address risks for SIs in patients identified as having PsA in routine clinical practice. Notably, the majority of available data regarding the impact of biologic therapies on SI risk has been directed at other inflammatory arthritides, such as RA [15, 16], and less so for patients with PsA [17]. Consequently, an unmet need remains to assess the risk for SI in the PsA population and potentially identify any differences between outcomes of exposure in real-world use that may exist. The Psoriasis Longitudinal Assessment and Registry (PSOLAR), an observational psoriasis registry with more than 12,000 patients, captures safety data among patients treated with biologics and/or systemic agents and can help address this real-world need.

The risk of SIs in patients with psoriasis from the PSOLAR registry has been previously reported [7]. In this report, we evaluated the incidence of SIs in a subpopulation of patients with self-reported PsA enrolled in the psoriasis PSOLAR registry [18], and specifically, among patients receiving biologic therapies for their psoriasis with concomitant PsA compared with patients receiving nonbiologic therapies. PSOLAR is an international, disease-based, observational study in which patients eligible for, or receiving, conventional systemic and biologic agents for the treatment of psoriasis are followed prospectively [18]. Predictors of time to first SI were also evaluated. This is the first modeled analysis of patients with self-reported PsA enrolled in PSOLAR, the largest psoriasis registry to date.

## Methods

### Patients and study design

The PSOLAR patient populations and study design have been previously reported [18]. Briefly, PSOLAR enrolled adult patients (> 18 years) receiving, or eligible to receive, treatment with biologics and/or conventional systemic agents for psoriasis [18, 19]. As of August 23, 2015, PSOLAR was fully enrolled with 12,090 patients.

In the current report, all patients had psoriasis, including a subset of which self-reported a diagnosis of PsA (*N* = 4315) [20]. Of the patients self-reporting PsA, a subset self-reported that their healthcare provider established a diagnosis of PsA (*N* = 1719), but with no further confirmation by the investigator [20]. Patients were enrolled at international dermatology sites including academic centers, hospitals, and community practices [18]. Demographic and psoriasis disease characteristics, medical, social, and family histories, and previous medication use were collected at each site using electronic case report forms (eCRF). Data were collected at site visits at baseline and every-6-months, except medical, social, and family histories, which were only collected at baseline.

PSOLAR is conducted in accordance with the International Conference on Harmonizing guidelines on Good Clinical Practices and the Declaration of Helsinki. An institutional review board or ethics committee (Goodwyn Institutional Review Board and Ontario Institutional Review Board) approved the registry protocol. All patients provided written informed consent. This study adheres to the STROBE guidelines for observational studies.

In this report, patients were stratified by exposure to specific biologics or non-biologics. Specifically, patients were evaluated using the following treatment cohorts: ustekinumab, infliximab, adalimumab, etanercept, all other biologics, non-biologic/methotrexate (MTX) and non-biologic/non-MTX. The specific tumor necrosis factor (TNF) inhibitors evaluated in this analysis (infliximab, adalimumab, and etanercept) were selected because they were the most prevalently used in the PSOLAR registry. The “all other biologics” cohort included patients receiving a biologic indicated for PsA (e.g., golimumab, certolizumab, secukinumab), that was being studied in PsA but not yet approved at the time (e.g., abatacept, brodalumab, ixekizumab), or that is no longer commercially available (e.g., efalizumab, alefacept). The “all other biologics” cohort has been removed from all tables presented in this report due to low numbers, but is still included in the total. Patients had received one of these agents when registered in PSOLAR or began treatment after PSOLAR registration. These patients may also have been exposed in the past to a different biologic, topical psoriasis therapy, or phototherapy. Previous use or current exposure was allowed for MTX, but not for other systemic immunomodulators. Non-biologic/non-MTX could have included, but were not limited to, cyclosporine, tacrolimus, mycophenolate mofetil, azathioprine, oral corticosteroids, and psoralen plus ultraviolet A, or ultraviolet B phototherapy. Patients in cohorts other than the non-biologic/non-MTX group were excluded if oral corticosteroids were used. This was done to allow for more fair comparisons and to prevent confounding the SI based on the risk of the biologic itself. For each comparison, the MTX-exposed patient cohort served as the reference group.

The cohorts described above were analyzed in three populations as follows. The overall population included prevalent (previous) users of biologics (receiving a biologic at the time of registry enrollment) and incident (new) users of biologics (starting a biologic at the time of, or after, enrollment). The incident biologic user subpopulation included only new users of a biologic at or after enrollment as defined above. Prior usage of any biologic other than the newly initiated biologic was allowed among incident users. The bionaive subpopulation included users who had never been exposed to any biologic. The primary analysis for this study was based on the overall population.

Index date (initiation of follow-up to record person years) for the cohorts was the date of registration into PSOLAR for those on current therapy OR the start date of therapy after registration for new users. Incident rates of SI were calculated based upon person years of exposure. Follow-up began on the index date until: 1) death; 2) withdrawal from the registry; 3) last data cut (August 2016); 4) 91 days after discontinuation of treatment; or 5) switching of treatment. Patients could not qualify for other treatment cohorts if switching of therapy occurred.

The exposure period for the new biologic users (incident user and bionaive subpopulations) started at the date of the first cohort-defining biologic dose on registry and ended at the earlier of 90 days after the last cohort-defining biologic dose, the date of switching to a different biologic, the date of starting other systemic immunomodulators, discontinuation from the registry, registry data cut (August 23, 2016), or death. The exposure period for prevalent biologic users started at registry enrollment and ended as described for the new biologic users. Only the first cohort-defining biologic was considered; biologic usage after switching was excluded.

Both non-biologic/MTX and non-biologic/non-MTX cohorts were defined the same for both prevalent and incident users. Exposure for the non-biologic/MTX was calculated from the later date of the enrollment date and the first MTX dose date and ended as the earlier of 90 days after the last MTX dose, the date of switching to other systemic immunomodulators, discontinuation from the registry, registry data cut (August 23, 2016), or death.

Exposure for the non-biologic/non-MTX starts from the later time of enrollment date and the start date of other immunomodulators on registry and ended at the earlier of 90 days after the last cohort defining drug dose, the date of switching to MTX, discontinuation from the registry, registry data cut (August 23, 2016), or death.

### Outcome measures

The outcome included the number of SI events, defined as serious adverse events (SAEs) classified as “Infections and Infestations” based on the Medical Dictionary for Regulatory Activities (MedDRA) coding system. SAEs are defined as any undesirable experience associated with the use of a medical product in a patient that may result in death, be life-threatening, lead to hospitalization or prolonged hospitalization, disability or permanent damage, congenital anomaly or birth defect, or require intervention to prevent permanent impairment or damage. All AEs were identified by the site investigator, collected through eCRF, and reviewed. Verbatim terms provided by sites are coded to MedDRA terms and are subject to verification by site monitors. All SAEs regardless of exposure are subject to verification by study personnel. Incomplete reports are queried for context to verify the event. This is performed by the study nurse and physician initially and cross-checked for completeness by the sponsor’s medical monitor. Queries are generated if the rationale/context of the reported event term is unclear. Despite monitoring of serious event documentation, source documents may not necessarily be obtainable for all diagnoses. Nonetheless, reporting, categorization and definition of serious infections is designed to be non-differential across all treatment cohorts since the study support team performs uniform follow-up on all cases. History of infections was defined as infections requiring a prescription medication within 3 years of enrollment. Rates of multiple infections and modeled analyses were based on time to first infection.

### Statistical analysis

**Fig. 1:**
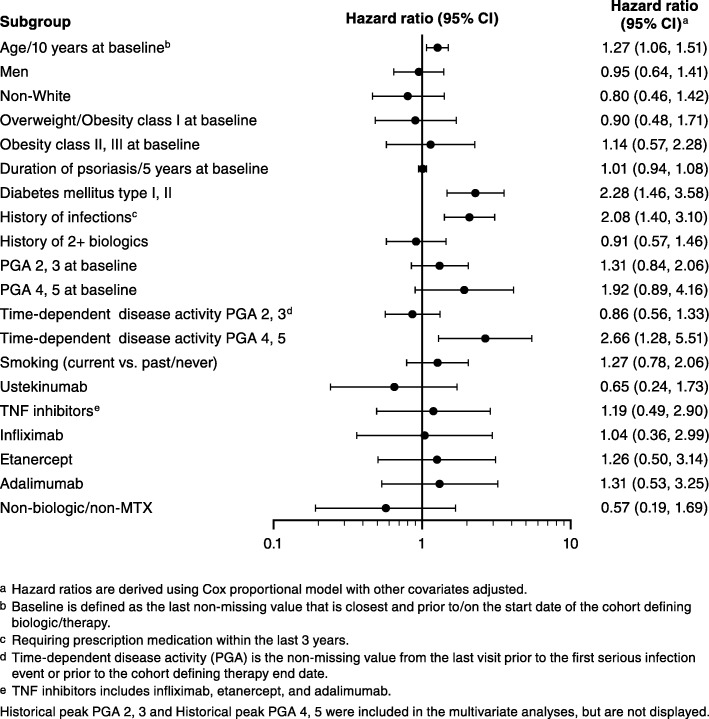
Predictors of time-to-first serious infection in PSOLAR psoriasis patients with self-reported PsA (overall population). CI, confidence interval; MTX, methotrexate; PGA, Physician’s Global Assessment; PsA, psoriatic arthritis; PSOLAR, Psoriasis Longitudinal Assessment and Registry; TNF, tumor necrosis factor

All patients from the PSOLAR registry at the time of this analysis (August 23, 2016) who self-reported having PsA were categorized into a treatment cohort based on the definitions mentioned above while excluding patients violating the rules. Baseline demographics and disease characteristics were summarized for each cohort group without imputation for missing values. Cumulative incidence rates for SI were calculated based on 100 patient-years. Cox regression models were used to identify predictors to first SI event and to estimate adjusted hazard ratios and 95% confidence intervals (CI) of SI event. Pre-determined covariates at baseline (defined as the last non-missing value that is closest and prior to/on the cohort start date) were included in the multivariate model (Fig. 1). In case of missing continuous covariates, they were imputed with population mean and the imputed values were used in the model.

## Results

### Baseline demographics and disease characteristics

A total of 12,090 patients with psoriasis were enrolled in the PSOLAR registry at the time of this analysis. Overall, approximately 36% (*n* = 4315) of the psoriasis patients in PSOLAR self-reported having PsA [7, 20].

The overall PsA population included a total of 2401 patients (7244 patient years [PY]), of which 628 had received ustekinumab; 1413 received TNF inhibitors; 258 received infliximab; 481 received etanercept, 674 received adalimumab, 98 received non-biologic/MTX, and 208 received non-biologic/non-MTX (Table 1). Biologic exposure for each group started at exposure to the first biologic therapy received on registry. The incident population included 1163 patients (3101 PY), and the bionaive population included 532 patients (1626 PY) (Table 2). In the overall population, the demographic and disease characteristics were generally comparable across therapeutic cohorts (Table 1). In general, the ratio of men to women was comparable, with a mean age of approximately 50 years. The majority of patients were white (83.3%) and overweight (84.6%), which typically are common findings in patients with psoriasis and PsA. All patients evaluated in this study self-reported a diagnosis of PsA, and high proportions of patients had a medical history of other comorbidities including: cardiovascular, psychiatric, endocrine, and pulmonary disorders (Table 1). Additionally, more than one-quarter of all patients in the overall PsA population (26.7%) had a history of infections, requiring prescription medication within the last 3 years, with the infliximab (32.6%) cohort having a slightly higher rate. Baseline characteristics for the incident (Additional file 1: Table S1) and bionaive (Additional file 2: Table S2) populations were generally comparable to those in the overall population.

**Table 1:** Demographic and disease characteristics of psoriasis patients with self-reported PsA in the PSOLAR registry: Overall population

	Ustekinumab (*N* = 628)	TNF inhibitors^a^ (*N* = 1413)	Infliximab (*N* = 258)	Etanercept (*N* = 481)	Adalimumab (*N* = 674)	Non-biologic /MTX^b^ (*N* = 98)	Non-biologic /non-MTX^c^ (*N* = 208)	All^d^ (N = 2401)
Age, years								
Mean ± SD	49.7 ± 12.6	50.4 ± 12.5	49.5 ± 12.6	51.1 ± 13.0	50.4 ± 12.0	55.6 ± 12.3	53.4 ± 14.3	50.8 ± 12.8
Sex								
Men	352 (56.1)	757 (53.6)	138 (53.5)	259 (53.8)	360 (53.4)	34 (34.7)	99 (47.6)	1272 (53.0)
Race								
White	527 (83.9)	1157 (81.9)	223 (86.4)	392 (81.5)	542 (80.4)	80 (81.6)	190 (91.3)	2000 (83.3)
Body mass index (BMI) (kg/m^2^)								
N	617	1372	245	471	656	93	203	2339
Mean ± SD	32.1 ± 7.0	31.7 ± 7.4	32.9 ± 8.4	31.1 ± 7.3	31.7 ± 7.1	30.8 ± 7.3	30.6 ± 6.9	31.7 ± 7.2
Obesity class								
N	617	1372	245	471	656	93	203	2339
Underweight-normal (BMI < 18.5–24.9)	80 (13.0)	209 (15.2)	29 (11.8)	81 (17.2)	99 (15.1)	24 (25.8)	41 (20.2)	359 (15.3)
Overweight-obesity class I (25.0–34.9)	353 (57.2)	779 (56.8)	136 (55.5)	268 (56.9)	375 (57.2)	43 (46.2)	110 (54.2)	1315 (56.2)
Obesity class II-III (35.0- ≥40)	184 (29.8)	384 (28.0)	80 (32.7)	122 (25.9)	182 (27.7)	26 (28.0)	52 (25.6)	665 (28.4)
Psoriasis disease characteristics								
Duration of psoriasis, years								
N	627	1400	256	477	667	97	208	2385
Mean ± SD	20.8 ± 13.0	19.0 ± 13.4	20.1 ± 13.8	18.0 ± 13.6	19.3 ± 13.2	17.4 ± 13.5	17.5 ± 15.4	19.3 ± 13.6
BSA involvement (%)								
N	616	1383	247	472	664	96	205	2353
Median	6.0	4.0	4.0	3.8	4.0	6.0	8.0	5.0
Baseline PGA score								
N	618	1386	249	474	663	96	205	2359
Mean ± SD	2.3 ± 1.2	1.9 ± 1.2	1.9 ± 1.2	1.9 ± 1.2	2.0 ± 1.3	2.4 ± 1.1	2.5 ± 1.1	2.1 ± 1.2
Medical history^e^								
PsA	628 (100.0)	1413 (100.0)	258 (100.0)	481 (100.0)	674 (100.0)	98 (100.0)	208 (100.0)	2401 (100.0)
Cardiovascular disease	272 (43.3)	605 (42.8)	121 (46.9)	214 (44.5)	270 (40.1)	43 (43.9)	100 (48.1)	1040 (43.3)
Psychiatric illness	158 (25.2)	344 (24.3)	69 (26.7)	117 (24.3)	158 (23.4)	21 (21.4)	49 (23.6)	584 (24.3)
Anxiety	84 (13.4)	196 (13.9)	40 (15.5)	69 (14.3)	87 (12.9)	10 (10.2)	31 (14.9)	327 (13.6)
Depression	116 (18.5)	236 (16.7)	46 (17.8)	78 (16.2)	112 (16.6)	14 (14.3)	36 (17.3)	413 (17.2)
Inflammatory bowel disease	16 (2.5)	37 (2.6)	8 (3.1)	11 (2.3)	18 (2.7)	4 (4.1)	5 (2.4)	64 (2.7)
Crohn’s disease	4 (0.6)	9 (0.6)	2 (0.8)	3 (0.6)	4 (0.6)	1 (1.0)	1 (0.5)	16 (0.7)
Ulcerative colitis	3 (0.5)	10 (0.7)	2 (0.8)	2 (0.4)	6 (0.9)	1 (1.0)	1 (0.5)	15 (0.6)
Indeterminate colitis	6 (1.0)	15 (1.1)	4 (1.6)	5 (1.0)	6 (0.9)	2 (2.0)	3 (1.4)	26 (1.1)
Pulmonary^f^	94 (15.0)	225 (15.9)	40 (15.5)	76 (15.8)	109 (16.2)	21 (21.4)	46 (22.1)	395 (16.5)
Hepatic	22 (3.5)	56 (4.0)	14 (5.4)	19 (4.0)	23 (3.4)	2 (2.0)	9 (4.3)	92 (3.8)
Skin cancer	28 (4.5)	88 (6.2)	14 (5.4)	38 (7.9)	36 (5.3)	6 (6.1)	15 (7.2)	144 (6.0)
Other cancers	17 (2.7)	48 (3.4)	9 (3.5)	18 (3.7)	21 (3.1)	13 (13.3)	14 (6.7)	95 (4.0)
Endocrine	135 (21.5)	315 (22.3)	60 (23.3)	113 (23.5)	142 (21.1)	26 (26.5)	52 (25.0)	539 (22.4)
Diabetes mellitus type I	8 (1.3)	23 (1.6)	6 (2.3)	10 (2.1)	7 (1.0)	1 (1.0)	7 (3.4)	39 (1.6)
Diabetes mellitus type II	90 (14.3)	181 (12.8)	35 (13.6)	62 (12.9)	84 (12.5)	15 (15.3)	33 (15.9)	325 (13.5)
Thyroid dysfunction	46 (7.3)	134 (9.5)	25 (9.7)	47 (9.8)	62 (9.2)	13 (13.3)	18 (8.7)	218 (9.1)
History of infections^g^	167 (26.6)	388 (27.5)	84 (32.6)	137 (28.5)	167 (24.8)	23 (23.5)	43 (20.7)	641 (26.7)
Social activity^e^								
Alcohol use, N	628	1413	258	481	674	98	208	2401
Never used	130 (20.7)	301 (21.3)	70 (27.1)	91 (18.9)	140 (20.8)	47 (48.0)	55 (26.4)	545 (22.7)
Current user	426 (67.8)	905 (64.0)	161 (62.4)	302 (62.8)	442 (65.6)	35 (35.7)	120 (57.7)	1521 (63.3)
Have used and stopped	72 (11.5)	207 (14.6)	27 (10.5)	88 (18.3)	92 (13.6)	16 (16.3)	33 (15.9)	335 (14.0)
Smoking, N	628	1412	257	481	674	98	208	2400
Never smoked	256 (40.8)	651 (46.1)	122 (47.5)	220 (45.7)	309 (45.8)	47 (48.0)	85 (40.9)	1059 (44.1)
Current smoker	147 (23.4)	287 (20.3)	61 (23.7)	85 (17.7)	141 (20.9)	24 (24.5)	53 (25.5)	521 (21.7)
Prior smoker, stopped	225 (35.8)	474 (33.6)	74 (28.8)	176 (36.6)	224 (33.2)	27 (27.6)	70 (33.7)	820 (34.2)

Data are n (%) unless otherwise indicated

The biologic user cohort includes patients who are on the cohort defining biologic at entry or start the biologic after entry; previous use or current exposure is allowed for MTX, but not for other systemic immunomodulators

*BMI* body mass index; *BSA* body surface area; *MTX* methotrexate; *PGA* Physician’s Global Assessment; *PsA* psoriatic arthritis; *PSOLAR* Psoriasis Longitudinal Assessment and Registry; *SD* standard deviation

^a^Tumor necrosis factor (TNF) inhibitors include infliximab, etanercept, and adalimumab

^b^ The non-biologic/MTX cohort includes patients who are on methotrexate at entry or start methotrexate during the registry and haven’t been exposed to other systemic immunomodulators previously or concurrently

^c^Non-biologic, non-MTX therapies may include, but are not limited to, cyclosporine, tacrolimus, mycophenolate mofetil, azathioprine, oral corticosteroids, systemic retinoids, psoralen plus ultraviolet (UV), or UVB phototherapy. The non-biologic/non-MTX cohort includes patients who are on other systemic immunomodulators (including cyclosporine, tacrolimus, mycophenolate mofetil, other immunomodulators, and oral corticosteroids) at entry or start other immunomodulators after registry and who haven’t been exposed to MTX previously or concurrently; patients who are on or receive only topical and/or phototherapy at/after registry entry are also in this cohort

^d^Includes “Other biologics” group (*n* = 54); data not shown

^e^Data were collected at baseline (defined as the last non-missing value that is closest and prior to/on the cohort start date)

^f^Includes sleep apnea, asthma, chronic obstructive pulmonary disorder, and pneumonitis

^g^History of infections is defined as infections within 3 years of enrollment and required a prescription medication

Obesity class based upon National Heart Lung and Blood Institute Obesity Education Initiative http://www.nhlbi.nih.gov/health/public/heart/obesity/lose_wt/bmi_dis.html

**Table 2:** Number of patient-years of follow-up by population and treatment cohort in psoriasis patients with self-reported PsA enrolled in PSOLAR

	Ustekinumab	TNF inhibitors^a^	Infliximab	Etanercept	Adalimumab	Non-biologic/MTX^b^	Non-biologic/non-MTX^c^	All^d^
Overall population								
N	628	1413	258	481	674	98	208	2401
Patient-years	2009	4101	756	1432	1913	366	694	7244
Duration of follow-up, years								
Mean ± SD	5.0 ± 1.9	4.9 ± 2.2	4.7 ± 2.3	4.8 ± 2.3	5.0 ± 2.1	4.2 ± 2.2	3.7 ± 2.6	4.8 ± 2.2
Median	5.16	5.16	4.70	5.16	5.35	4.32	3.50	5.11
Incident population								
N	326	486	64	130	292	98	208	1163
Patient-years	904	1082	125	291	665	366	694	3101
Duration of follow-up, years								
Mean ± SD	5.4 ± 1.9	5.4 ± 2.0	5.3 ± 2.0	5.5 ± 2.0	5.4 ± 2.0	4.2 ± 2.2	3.7 ± 2.6	5.0 ± 2.2
Median	5.71	5.61	5.67	5.64	5.59	4.32	3.50	5.40
Bionaive population								
N	56	167	14	72	81	98	208	532
Patient-years	144	419	35	180	204	366	694	1626
Duration of follow-up, years								
Mean ± SD	4.4 ± 2.0	5.2 ± 2.0	4.6 ± 1.8	5.6 ± 2.2	4.8 ± 1.9	4.2 ± 2.2	3.7 ± 2.6	4.4 ± 2.3
Median	4.66	5.52	4.39	6.17	5.14	4.32	3.50	4.70

The biologic user cohort includes patients who are on the cohort defining biologic at entry or start the biologic after entry; previous use or current exposure is allowed for MTX, but not for other systemic immunomodulators

*MTX* methotrexate; PsA, psoriatic arthritis; PSOLAR, Psoriasis Longitudinal Assessment and Registry; SD, standard deviation

^a^Tumor necrosis factor (TNF) inhibitors include infliximab, etanercept, and adalimumab

^b^The non-biologic/MTX cohort includes patients who are receiving MTX at entry or start methotrexate during the registry and haven’t been exposed to other systemic immunomodulators previously or concurrently

^c^Non-biologic, non-MTX therapies may include, but are not limited to, cyclosporine, tacrolimus, mycophenolate mofetil, azathioprine, oral corticosteroids, systemic retinoids, psoralen plus ultraviolet (UV), or UVB phototherapy. The non-biologic/non-MTX cohort includes patients who are receiving other systemic immunomodulators (including cyclosporine, tacrolimus, mycophenolate mofetil, other immunomodulators, and oral corticosteroids) at entry or start other immunomodulators after registry and who haven’t been exposed to MTX previously or concurrently; patients who receive only topical and/or phototherapy at/after registry entry are also in this cohort

^d^Includes “Other biologics” group (*n* = 54); data not shown

### Prior medication use

In the overall population, patients in the non-biologic/non-MTX cohort had the greatest use of systemic steroids (23.1%) (Table 3). MTX was the most commonly used immunomodulator among all biologic user cohorts, with the greatest reported used (60.1%) in the infliximab cohort. As expected among psoriasis patients, topical therapies were used in the majority of patients. Overall, 77.8% of all patients had a history of biologics use, with 41.5% having used only one biologic (Table 3).

**Table 3:** Prior medication use at enrollment in psoriasis patients with self-reported PsA enrolled in PSOLAR; Overall population

	Ustekinumab (N = 628)	TNF inhibitors^a^ (N = 1413)	Infliximab (N = 258)	Etanercept (N = 481)	Adalimumab (N = 674)	Non-biologic /MTX^b^ (N = 98)	Non-biologic /non-MTX^c^ (N = 208)	All^d^ (*N* = 2401)
Systemic steroids^e^	0 (0.0)	0 (0.0)	0 (0.0)	0 (0.0)	0 (0.0)	0 (0.0)	48 (23.1)	48 (2.0)
Immunomodulators	259 (41.2)	568 (40.2)	155 (60.1)	171 (35.6)	242 (35.9)	81 (82.7)	11 (5.3)	947 (39.4)
Methotrexate	259 (41.2)	568 (40.2)	155 (60.1)	171 (35.6)	242 (35.9)	81 (82.7)	0 (0.0)	936 (39.0)
Cyclosporine	0 (0.0)	0 (0.0)	0 (0.0)	0 (0.0)	0 (0.0)	0 (0.0)	6 (2.9)	6 (0.2)
Oral tacrolimus	0 (0.0)	0 (0.0)	0 (0.0)	0 (0.0)	0 (0.0)	0 (0.0)	0 (0.0)	0 (0.0)
Mycophenolate mofetil	0 (0.0)	0 (0.0)	0 (0.0)	0 (0.0)	0 (0.0)	0 (0.0)	1 (0.5)	1 (< 0.1)
Other immunomodulators^e^	0 (0.0)	0 (0.0)	0 (0.0)	0 (0.0)	0 (0.0)	0 (0.0)	5 (2.4)	5 (0.2)
Topical therapy	609 (97.0)	1381 (97.7)	249 (96.5)	472 (98.1)	660 (97.9)	96 (98.0)	194 (93.3)	2332 (97.1)
Topical steroids	602 (95.9)	1352 (95.7)	244 (94.6)	462 (96.0)	646 (95.8)	94 (95.9)	187 (89.9)	2285 (95.2)
Phototherapy	376 (59.9)	670 (47.4)	142 (55.0)	214 (44.5)	314 (46.6)	37 (37.8)	90 (43.3)	1200 (50.0)
Psoralens + UVA	111 (17.7)	210 (14.9)	56 (21.7)	56 (11.6)	98 (14.5)	13 (13.3)	14 (6.7)	358 (14.9)
Biologics	572 (91.1)	1246 (88.2)	244 (94.6)	409 (85.0)	593 (88.0)	0 (0.0)	0 (0.0)	1869 (77.8)
Number of biologics used	628	1413	258	481	674	98	208	2401
0	56 (8.9)	167 (11.8)	14 (5.4)	72 (15.0)	81 (12.0)	98 (100.0)	208 (100.0)	532 (22.2)
1	207 (33.0)	769 (54.4)	90 (34.9)	318 (66.1)	361 (53.6)	0 (0.0)	0 (0.0)	997 (41.5)
2	215 (34.2)	342 (24.2)	94 (36.4)	69 (14.3)	179 (26.6)	0 (0.0)	0 (0.0)	569 (23.7)
3	110 (17.5)	112 (7.9)	48 (18.6)	18 (3.7)	46 (6.8)	0 (0.0)	0 (0.0)	234 (9.7)
4+	40 (6.4)	23 (1.6)	12 (4.7)	4 (0.8)	7 (1.0)	0 (0.0)	0 (0.0)	69 (2.9)

Data are n (%) unless otherwise indicated

The biologic user cohort includes patients who are on the cohort defining biologic at entry or start the biologic after entry; previous use or current exposure is allowed for MTX, but not for other systemic immunomodulators

*MTX* methotrexate; *PsA* psoriatic arthritis; *PSOLAR* Psoriasis Longitudinal Assessment and Registry; *UVA* ultraviolet A

^a^ Tumor necrosis factor (TNF) inhibitors includes infliximab, etanercept, and adalimumab

^b^The non-biologic/MTX cohort includes patients who are receiving MTX at entry or start methotrexate during the registry and haven’t been exposed to other systemic immunomodulators previously or concurrently

^c^Non-biologic, non-MTX therapies may include, but are not limited to, cyclosporine, tacrolimus, mycophenolate mofetil, azathioprine, oral corticosteroids, systemic retinoids, psoralen plus ultraviolet (UV), or UVB phototherapy. The non-biologic/non-MTX cohort includes patients who are receiving other systemic immunomodulators (including cyclosporine, tacrolimus, mycophenolate mofetil, other immunomodulators, and oral corticosteroids) at entry or start other immunomodulators after registry and who haven’t been exposed to MTX previously or concurrently; patients who receive only topical and/or phototherapy at/after registry entry are also in this cohort

^d^Includes “Other biologics” group (n = 54); data not shown

^e^Prohibited per exclusion criteria

### Incidence rates of SI

In the overall population, among patients receiving specific biologics, the rates of SIs were highest in the etanercept (2.58), infliximab (2.12), and adalimumab (1.99) cohorts, respectively (Table 4). The ustekinumab cohort had a numerically lower incidence rate (1.00) among the biologics tested individually and when compared with the rates reported in the two non-biologics cohorts. No SI events were reported in the “all other biologics” cohort (data not shown). The incident rates of SIs in the incident user were similar to those reported in the overall population; however, the number of patients in the bionaive population was too low to compare with the overall population (Table 4).

**Table 4:** Cumulative incidence rates of serious infections per 100 patient-years in psoriasis patients with self-reported PsA enrolled in PSOLAR; By cohort

	Ustekinumab	TNF inhibitors^a^	Infliximab	Etanercept	Adalimumab	Non-biologic/MTX^b^	Non-biologic/non-MTX^c^	All^d^
Overall population								
N	628	1413	258	481	674	98	208	2401
PY	2009	4101	756	1432	1913	366	694	7244
Serious infections and infestations	1.00 [20]	2.22 [91]	2.12 [16]	2.58 [37]	1.99 [38]	3.01 [11]	2.31 [16]	1.91 [138]
Incident population								
N	326	486	64	130	292	98	208	1163
PY	904	1082	125	291	665	366	694	3101
Serious infections and infestations	0.88 [8]	2.50 [27]	0.80 [1]	3.09 [9]	2.56 [17]	3.01 [11]	2.31 [16]	2.00 [62]
Bionaive population								
N	56	167	14	72	81	98	208	532
PY	144	419	35	180	204	366	694	1626
Serious infections and infestations	1.39 [2]	1.91 [8]	0.00 [0]	3.33 [6]	0.98 [2]	3.01 [11]	2.31 [16]	2.28 [37]

Data are incidence rate [n]

The biologic user cohort includes patients who are on the cohort defining biologic at entry or start the biologic after entry; previous use or current exposure is allowed for MTX, but not for other systemic immunomodulators

The incidence of adverse events is reported as rate of adverse events per 100 PY. Number of PY is defined as the number of years exposed to the cohort defining medication. Exposure starts from first exposure to a medication on\during registry participation and ends at the earlier of the date of reference end date, initiating another biologic/medication, 90 days after the last dose of the cohort defining treatment, or the date of the annual data cutoff (23AUG2016), whichever is sooner.

PY = number of days exposed / 365.25

*MTX* methotrexate; *PsA* psoriatic arthritis; *PSOLAR* Psoriasis Longitudinal Assessment and Registry; *PY* patient-years

^a^Tumor necrosis factor (TNF) inhibitors include infliximab, etanercept, and adalimumab

^b^The non-biologic/MTX cohort includes patients who are receiving MTX at entry or start methotrexate during the registry and haven’t been exposed to other systemic immunomodulators previously or concurrently

^c^Non-biologic, non-MTX therapies may include, but are not limited to, cyclosporine, tacrolimus, mycophenolate mofetil, azathioprine, oral corticosteroids, systemic retinoids, psoralen plus ultraviolet (UV), or UVB phototherapy. The non-biologic/non-MTX cohort includes patients who are receiving other systemic immunomodulators (including cyclosporine, tacrolimus, mycophenolate mofetil, other immunomodulators, and oral corticosteroids) at entry or start other immunomodulators after registry and who haven’t been exposed to MTX previously or concurrently; patients who receive only topical and/or phototherapy at/after registry entry are also in this cohort

^d^Includes “Other biologics” group (n = 54); data not shown

In the overall population, the incidence rate of SI was 1.91 (95% CI: 1.60, 2.25; *n* = 138) per 7244 PY of exposure (Table 5). The SIs listed by type of infection that occurred at least two or more times across treatment cohorts were captured in Table 5. The highest incidences rate of SI among biologic users were observed in the etanercept (2.58; *n* = 37), TNF inhibitors (2.22; *n* = 91), and infliximab (2.12; *n* = 16) cohorts, while the ustekinumab cohort had the lowest incidence rate of SI (1.00; *n* = 20). Cellulitis (*n* = 25) and pneumonia (*n* = 24) were the most commonly reported SIs among all patients, with the most cases of cellulitis (*n* = 22) and pneumonia (*n* = 17) both reported in the TNF inhibitors cohort (Table 5).

**Table 5:** Cumulative incidence of serious infections per 100 patient-years by type of infection in psoriasis patients with self-reported PsA enrolled in PSOLAR; Overall population

	Ustekinumab (*N* = 2009 PY)	TNF inhibitors^a^ (*N* = 4010 PY)	Infliximab (*N* = 756 PY)	Etanercept (*N* = 1432 PY)	Adalimumab (*N* = 1913 PY)	Non-biologic /MTX^b^ (*N* = 366 PY)	Non-biologic /non-MTX^c^ (*N* = 694 PY)	All^d^ (*N* = 7244 PY)
Serious infections^e^	1.00 [20]	2.22 [91]	2.12 [16]	2.58 [37]	1.99 [38]	3.01 [11]	2.31 [16]	1.91 [138]
Appendicitis, perforated	0.05 [1]	0.05 [2]	0.00 [0]	0.00 [0]	0.10 [2]	0.00 [0]	0.14 [1]	0.06 [4]
Arthritis, infectious	0.00 [0]	0.02 [1]	0.00 [0]	0.07 [1]	0.00 [0]	0.00 [0]	0.14 [1]	0.03 [2]
Cellulitis	0.05 [1]	0.54 [22]	0.66 [5]	0.84 [12]	0.26 [5]	0.55 [2]	0.00 [0]	0.35 [25]
*Clostridium difficile* colitis	0.00 [0]	0.00 [0]	0.00 [0]	0.00 [0]	0.00 [0]	0.27 [1]	0.29 [2]	0.04 [3]
Clostridium difficile infection	0.00 [0]	0.02 [10]	0.13 [1]	0.00 [0]	0.00 [0]	0.27 [1]	0.00 [0]	0.03 [2]
Device-related infection	0.00 [0]	0.07 [3]	0.13 [1]	0.07 [1]	0.05 [1]	0.00 [0]	0.00 [0]	0.04 [3]
Diverticulitis	0.05 [1]	0.17 [7]	0.00 [0]	0.35 [5]	0.10 [2]	0.00 [0]	0.14 [1]	0.12 [9]
Gastroenteritis	0.00 [0]	0.07 [3]	0.00 [0]	0.07 [1]	0.10 [2]	0.27 [1]	0.14 [1]	0.07 [5]
Herpes zoster	0.00 [0]	0.07 [3]	0.00 [0]	0.14 [2]	0.05 [1]	0.00 [0]	0.00 [0]	0.04 [3]
Pneumonia	0.15 [3]	0.41 [17]	0.26 [2]	0.49 [7]	0.42 [8]	0.55 [2]	0.29 [2]	0.33 [24]
Postoperative wound infection	0.00 [0]	0.00 [0]	0.00 [0]	0.00 [0]	0.00 [0]	0.27 [1]	0.14 [1]	0.03 [2]
Acute pyelonephritis	0.00 [0]	0.02 [1]	0.00 [0]	0.00 [0]	0.05 [1]	0.00 [0]	0.14 [1]	0.03 [2]
Sepsis	0.05 [1]	0.05 [2]	0.00 [0]	0.07 [1]	0.05 [1]	0.00 [0]	0.00 [0]	0.04 [3]
Septic shock	0.00 [0]	0.05 [2]	0.13 [1]	0.07 [1]	0.00 [0]	0.27 [1]	0.00 [0]	0.04 [3]
Urinary tract infection	0.00 [0]	0.07 [3]	0.00 [0]	0.14 [2]	0.05 [1]	0.00 [0]	0.00 [0]	0.04 [3]
Urosepsis	0.05 [1]	0.00 [0]	0.00 [0]	0.00 [0]	0.00 [0]	0.00 [0]	0.29 [2]	0.04 [3]

Data are incidence rate [n]

The biologic user cohort includes patients who are on the cohort defining biologic at entry or start the biologic after entry; previous use or current exposure is allowed for MTX, but not for other systemic immunomodulators

PY = number of days exposed / 365.25

The following opportunistic infections (OIs) were reported (incidence rate [n]): coccidioidomycosis (ustekinumab: 0.05 [1]); herpes zoster (etanercept: 0.14 [2]; adalimumab (0.05 [1]); herpes zoster disseminated (infliximab: 0.13 [1]); infectious mononucleosis (infliximab: 0.13 [1]); pneumonia legionella (adalimumab: 0.05 [1]). No OIs were reported in the non-biologics/MTX and non-biologics/non-MTX groups

*MTX* methotrexate; *PsA* psoriatic arthritis; *PSOLAR* Psoriasis Longitudinal Assessment and Registry; *PY* patient-years

^a^Tumor necrosis factor (TNF) inhibitors include infliximab, etanercept, and adalimumab

^b^The non-biologic/MTX cohort includes patients who are receiving MTX at entry or start methotrexate during the registry and haven’t been exposed to other systemic immunomodulators previously or concurrently

^c^Non-biologic, non-MTX therapies may include, but are not limited to, cyclosporine, tacrolimus, mycophenolate mofetil, azathioprine, oral corticosteroids, systemic retinoids, psoralen plus ultraviolet (UV), or UVB phototherapy. The non-biologic/non-MTX cohort includes patients who are receiving other systemic immunomodulators (including cyclosporine, tacrolimus, mycophenolate mofetil, other immunomodulators, and oral corticosteroids) at entry or start other immunomodulators after registry and who haven’t been exposed to MTX previously or concurrently; patients who receive only topical and/or phototherapy at/after registry entry are also in this cohort

^d^Includes “Other biologics” group (*n* = 54); data not shown

^e^Serious infections (SIs) occurred at least 2 times across treatment cohorts. SIs as classified by the investigator are included in this analysis. The incidence of SIs is reported as rate of SIs per 100 PY. Number of PY is defined as the number of years exposed to the cohort defining medication. Exposure starts from first exposure to a medication on\during registry participation and ends at the earlier of the date of reference end date, initiating another biologic/medication, 90 days after the last dose of the cohort defining treatment, or the date of the annual data cutoff (23AUG2016), whichever is sooner

Multivariate analysis was used to determine predictors of time to first SI. Of the variables tested for the overall population, age, time-dependent disease activity PGA of 4, 5 (PGA scores of 4 or 5 at the time closest to the reported event), history of infection, and diabetes were associated (*p* < 0.05) with an increased risk for SI (Fig. 1). No increased risk for SI was observed with ustekinumab or non-biologic/non-MTX use. Findings were similar in the incident and bionaive populations; however, the number of patients in these cohorts was low and the 95% CIs were wide (data not shown).

## Discussion

Patients with PsA who are receiving biologics may have an elevated risk for developing SI. In this report, we used the PSOLAR psoriasis registry to evaluate 4315 psoriasis patients with self-reported PsA. This is the first modeled analysis of psoriasis patients with self-reported PsA enrolled in PSOLAR, the largest psoriasis registry to date.

It has been reported in several studies that TNF inhibitors are associated with an increased risk of SI in patients with psoriasis [2, 4–7] and in patients with PsA or RA [8, 21, 22]. Our results confirmed these findings among PSOLAR psoriasis patients self-reporting PsA [20]. These results are consistent with those previously reported for the overall PSOLAR population of patients with psoriasis, in which a higher risk of serious infections with the monoclonal TNF inhibitors compared with non-methotrexate and non-biologic therapies was found, with no increased risk observed with ustekinumab or etanercept [7]. With no adjustment, although not statistically significant, the SI rate was numerically higher in patients receiving TNF inhibitors and non-biologics compared with patients receiving ustekinumab. After adjusting for potential confounders, the risk of SI for patients receiving ustekinumab or non-biologic/non-MTX was lower than MTX, while the risk of all TNF inhibitors was higher than MTX. However, none of the differences reached statistical significance (*p* < 0.05). The incidence rates of SIs in the non-biologic/non-MTX group were high (2.31); however, this cohort included patients who had received systemic corticosteroids. Rates of SIs in the overall population were similar to those reported in the incident population, but differed compared with the bionaive population in which the number of patients was low. Likewise, rates of SIs reported here were similar to those in PsA clinical trials with biologics [8, 17, 23–25].

Currently, an unmet need remains for the treatment of patients with PsA. Most of the clinical and registry data available regarding safety pertains to patients with psoriasis or RA. Therefore, PSOLAR allows for the evaluation of a large population of patients with self-reported PsA, with greater than 12,000 patients registered, for a median of 5.11 years, and a total of 7244 PY of follow-up.

Patients with PsA often have other comorbidities thought to be associated with risk of infections. Several of the variables evaluated in this report, including age, time-dependent PGA 4, 5, history of infection, and diabetes were associated with a significantly increased risk for SI (*p* < 0.05). Interestingly, these variables were identical to those previously reported as being associated with SIs in the overall PSOLAR psoriasis population [7], thereby supporting our findings of psoriasis patients with self-reported PsA.

There are several limitations to consider here, as with any observational study. Treatment selection, recall, and reporting biases may have occurred, as these data are from a registry and not a clinical trial in which patients are randomized to treatment. PSOLAR longitudinally follows psoriasis patients; therefore, the subset of psoriasis patients with self-reported PsA could reflect potential treatment bias and may not reflect patients in the general rheumatology population. This is not a Classification Criteria for Psoriatic Arthritis defined PsA population, although up to approximately 30% of psoriasis patients may also develop concomitant PsA [1, 3]. The fact that these patients also had psoriasis may have affected their medical history and influenced their therapy choices, thereby contributing to treatment bias. The increased use of biologics in this PsA subpopulation may be due to these patients having more active/severe psoriasis. Prior use of biologics was accounted for in the model; however, additional risks from previous therapies may not have been addressed. Additionally, patients in the non-biologic/non-MTX group only answered “yes” or “no” to the question of if they had received oral corticosteroids, with no details of dosing. In addition, these patients self-reported having PsA by completing an eCRF, with no further confirmation of their diagnosis by the investigator. Finally, some statistical differences were noted in patient characteristics across biologic cohorts at entry into the registry. Possible confounding characteristics were included in the multivariate model; however, there may be other characteristics that were not collected and therefore not accounted for in the model.

According to recent guidelines for the treatment of PsA, serious infections were chosen as one of the critical outcomes for comparisons between therapies and are noted to be one of the greatest concerns for patients and physicians when choosing among the currently available therapies [26]. The findings presented here could potentially inform and assist health care professionals when selecting an appropriate treatment option for their patients with PsA.

## Conclusions

This analysis evaluated the incidence of SIs among the 2401 psoriasis patients with self-reported PsA in the overall population of the PSOLAR registry. Our results showed that rates of SIs were numerically higher among the self-reported PsA patients receiving TNF inhibitors (infliximab, adalimumab, or etanercept), but not in patients receiving ustekinumab; however, these differences were not statistically significant. Factors including increasing age, time-dependent PGA 4, 5, history of infection, and diabetes were also associated with an increased risk for SI in psoriasis patients with PsA. Continued follow-up of these PsA patients in the ongoing PSOLAR registry will provide additional safety information regarding patients with PsA in general.

## Supplementary information

**Additional file 1: Table S1.** Demographic and disease characteristics of psoriasis patients with self-reported PsA in the PSOLAR registry: Incident population.

**Additional file 2: Table S2.** Demographic and disease characteristics of psoriasis patients with self-reported PsA in the PSOLAR registry: Bionaive population.

## Data Availability

The datasets generated and/or analyzed during the current study are not currently publicly available because the registry is still ongoing, but will be available by request or at clinicaltrials.gov (NCT00508547) when the registry concludes.

## Abbreviations

AEs: Adverse events
CI: Confidence interval
eCRF: Electronic case report forms
MedDRA: Medical Dictionary for Regulatory Activities
MTX: Methotrexate
PsA: Psoriatic arthritis
PSOLAR: Psoriasis Longitudinal Assessment and Registry
PY: Patient years
RA: Rheumatoid arthritis
SAEs: Serious adverse events
SIs: Serious infections
TNF: Tumor necrosis factor
