# Supplementary material for: Serious infections in patients with self-reported psoriatic arthritis from the Psoriasis Longitudinal Assessment and Registry (PSOLAR) treated with biologics
Source: BMC Rheumatol. 2019 Nov 28;3:52. doi: 10.1186/s41927-019-0094-3 (PMC6882230; doi:10.1186/s41927-019-0094-3)
Supplement: Supplementary file 1 — Additional file 1: Table S1. Demographic and disease characteristics of psoriasis patients with self-reported PsA in the PSOLAR registry: Incident population. [file 41927_2019_94_MOESM1_ESM.docx]

**Table S1. Demographic and disease characteristics of psoriasis patients with self-reported PsA in the PSOLAR registry: Incident population**

|  | Ustekinumab (N=326) | TNF inhibitors^a^  (N=486) | Infliximab (N=64) | Etanercept (N=130) | Adalimumab (N=292) | Non-biologic /MTX^b^ (N=98) | Non-biologic /non-MTX^c^ (N=208) | All^d^ (N=1163) |
| --- | --- | --- | --- | --- | --- | --- | --- | --- |
| Age, years |  |  |  |  |  |  |  |  |
| Mean ± SD | 49.6 ± 13.4 | 49.3 ± 12.5 | 49.8 ± 12.1 | 50.5 ± 13.1 | 50.4 ± 12.6 | 55.6 ± 12.3 | 53.4 ± 14.3 | 51.2 ± 13.3 |
| Sex |  |  |  |  |  |  |  |  |
| Men | 171 (52.5) | 256 (52.7) | 38 (59.4) | 62 (47.7) | 156 (53.4) | 34 (34.7) | 99 (47.6) | 583 (50.1) |
| Race |  |  |  |  |  |  |  |  |
| White | 264 (81.0) | 399 (82.1) | 58 (90.6) | 105 (80.8) | 236 (80.8) | 80 (81.6) | 190 (91.3) | 972 (83.6) |
| BMI (kg/m^2^) |  |  |  |  |  |  |  |  |
| N | 320 | 479 | 62 | 128 | 290 | 93 | 203 | 1141 |
| Mean ± SD | 32.1 ± 7.0 | 32.2 ± 7.6 | 33.5 ± 7.8 | 32.5 ± 8.4 | 31.9 ± 7.4 | 30.8 ± 7.3 | 30.6 ± 6.9 | 31.9 ± 7.3 |
| Obesity class |  |  |  |  |  |  |  |  |
| Underweight-normal (BMI <18.5-24.9) | 39 (12.2) | 77 (16.1) | 5 (8.1) | 23 (18.0) | 49 (16.9) | 24 (25.8) | 41 (20.2) | 184 (16.2) |
| Overweight-obesity class I (25.0-34.9) | 189 (59.1) | 261 (54.5) | 34 (54.9) | 66 (51.6) | 154 (53.1) | 43 (46.3) | 110 (54.2) | 621 (54.4) |
| Obesity class II-III (35.0- ≥40) | 92 (28.8) | 141 (29.4) | 23 (37.1) | 39 (30.5) | 87 (30.0) | 26 (28.0) | 52 (25.6) | 336 (29.5) |
|  |  |  |  |  |  |  |  |  |
| **Psoriasis disease characteristics** |  |  |  |  |  |  |  |  |
| Duration of psoriasis, years |  |  |  |  |  |  |  |  |
| N | 325 | 482 | 63 | 130 | 289 | 97 | 208 | 1157 |
| Mean ± SD | 20.3 ± 13.2 | 18.2 ± 13.6 | 19.1 ± 13.5 | 15.2 ± 13.7 | 19.4 ± 13.5 | 17.4 ± 13.5 | 17.5 ± 15.4 | 18.7 ± 13.9 |
|  |  |  |  |  |  |  |  |  |
| BSA involvement (%) |  |  |  |  |  |  |  |  |
| N | 320 | 483 | 63 | 129 | 291 | 96 | 205 | 1149 |
| Median | 10.0 | 8.0 | 10.0 | 10.0 | 6.0 | 6.0 | 8.0 | 8.0 |
|  |  |  |  |  |  |  |  |  |
| Baseline PGA score |  |  |  |  |  |  |  |  |
| N | 322 | 484 | 63 | 130 | 291 | 96 | 205 | 1152 |
| Mean ± SD | 2.6 ± 1.1 | 2.5 ± 1.2 | 2.8 ± 1.1 | 2.4 ± 1.2 | 2.4 ± 1.2 | 2.4 ± 1.1 | 2. 5 ± 1.1 | 2.5 ± 1.2 |
|  |  |  |  |  |  |  |  |  |
| **Medical history**^e^ |  |  |  |  |  |  |  |  |
| Psoriatic arthritis | 326 (100.0) | 486 (100.0) | 64 (100.0) | 130 (100.0) | 292 (100.0) | 98 (100.0) | 208 (100.0) | 1163 (100.0) |
|  |  |  |  |  |  |  |  |  |
| Cardiovascular disease | 137 (42.0) | 206 (42.4) | 34 (53.1) | 54 (41.5) | 118 (40.4) | 43 (43.9) | 100 (48.1) | 503 (43.3) |
| Psychiatric illness | 97 (29.8) | 128 (26.3) | 23 (35.9) | 42 (32.3) | 63 (21.6) | 21 (21.4) | 49 (23.6) | 304 (26.1) |
| Anxiety | 55 (16.9) | 77 (15.8) | 14 (21.9) | 28 (21.5) | 35 (12.0) | 10 (10.2) | 31 (14.9) | 178 (15.3) |
| Depression | 70 (21.5) | 96 (19.8) | 16 (25.0) | 29 (22.3) | 51 (17.5) | 14 (14.3) | 36 (17.3) | 224 (19.3) |
| Inflammatory bowel disease | 7 (2.1) | 14 (2.9) | 1 (1.6) | 3 (2.3) | 10 (3.4) | 4 (4.1) | 5 (2.4) | 31 (2.7) |
| Crohn's disease | 2 (0.6) | 2 (0.4) | 0 (0.0) | 0 (0.0) | 2 (0.7) | 1 (1.0) | 1 (0.5) | 7 (0.6) |
| Ulcerative colitis | 3 (0.9) | 4 (0.8) | 0 (0.0) | 2 (1.5) | 2 (0.7) | 1 (1.0) | 1 (0.5) | 9 (0.8) |
| Indeterminate colitis | 1 (0.3) | 7 (1.4) | 1 (1.6) | 1 (0.8) | 5 (1.7) | 2 (2.0) | 3 (1.4) | 13 (1.1) |
| Pulmonary | 52 (16.0) | 78 (16.0) | 6 (9.4) | 21 (16.2) | 51 (17.5) | 21 (21.4) | 46 (22.1) | 205 (17.6) |
| Hepatic | 15 (4.6) | 17 (3.5) | 1 (1.6) | 4 (3.1) | 12 (4.1) | 2 (2.0) | 9 (4.3) | 45 (3.9) |
| Skin cancer | 15 (4.6) | 28 (5.8) | 4 (6.3) | 10 (7.7) | 14 (4.8) | 6 (6.1) | 15 (7.2) | 70 (6.0) |
| Other cancers | 11 (3.4) | 12 (2.5) | 0 (0.0) | 5 (3.8) | 7 (2.4) | 13 (13.3) | 14 (6.7) | 52 (4.5) |
| Endocrine | 78 (23.9) | 104 (21.4) | 15 (23.4) | 33 (25.4) | 56 (19.2) | 26 (26.5) | 52 (25.0) | 270 (23.2) |
| Diabetes mellitus type I | 7 (2.1) | 9 (1.9) | 1 (1.6) | 3 (2.3) | 5 (1.7) | 1 (1.0) | 7 (3.4) | 24 (2.1) |
| Diabetes mellitus type II | 52 (16.0) | 55 (11.3) | 7 (10.9) | 20 (15.4) | 28 (9.6) | 15 (15.3) | 33 (15.9) | 160 (13.8) |
| Thyroid dysfunction | 27 (8.3) | 46 (9.5) | 8 (12.5) | 10 (7.7) | 28 (9.6) | 13 (13.3) | 18 (8.7) | 111 (9.5) |
| History of infections^f^ | 101 (31.0) | 136 (28.0) | 25 (39.1) | 41 (31.5) | 70 (24.0) | 23 (23.5) | 43 (20.7) | 319 (27.4) |
|  |  |  |  |  |  |  |  |  |
| **Social activity**^e^ |  |  |  |  |  |  |  |  |
| Alcohol use | 326 | 486 | 64 | 130 | 292 | 98 | 208 | 1163 |
| Never used | 80 (24.5) | 120 (24.7) | 20 (31.3) | 28 (21.5) | 72 (24.7) | 47 (48.0) | 55 (26.4) | 312 (26.8) |
| Current user | 200 (61.3) | 290 (59.7) | 38 (59.4) | 70 (53.8) | 182 (62.3) | 35 (35.7) | 120 (57.7) | 674 (58.0) |
| Have used and stopped | 46 (14.1) | 76 (15.6) | 6 (9.4) | 32 (24.6) | 38 (13.0) | 16 (16.3) | 33 (15.9) | 177 (15.2) |
|  |  |  |  |  |  |  |  |  |
| Smoking |  |  |  |  |  |  |  |  |
| Never smoked | 132 (40.5) | 217 (44.7) | 31 (48.4) | 56 (43.1) | 130 (44.5) | 47 (48.0) | 85 (40.9) | 498 (42.8) |
| Current smoker | 81 (24.8) | 115 (23.7) | 17 (26.6) | 26 (20.0) | 72 (24.7) | 24 (24.5) | 53 (25.5) | 282 (24.2) |
| Prior smoker, stopped | 113 (34.7) | 154 (31.7) | 16 (25.0) | 48 (36.9) | 90 (30.8) | 27 (27.6) | 70 (33.7) | 383 (32.9) |
| Data are n (%) unless otherwise indicated.  BMI, body mass index; BSA, body surface area; MTX, methotrexate; PsA, psoriatic arthritis; PGA, Physician’s Global Assessment; PSOLAR, Psoriasis Longitudinal Assessment and Registry; SD, standard deviation  The biologic user cohort includes patients who are on the cohort defining biologic at entry or start the biologic after entry; previous use or current exposure is allowed for MTX, but not for other systemic immunomodulators.  ^a^ Tumor necrosis factor (TNF) inhibitors includes infliximab, etanercept and adalimumab.  ^b^ The non-biologic/MTX cohort includes patients who are receiving MTX at entry or start methotrexate during the registry and haven't been exposed to other systemic immunomodulators previously or concurrently.  ^c^ Non‐biologic, non-MTX therapies may include, but are not limited to cyclosporine, tacrolimus, mycophenolate mofetil, azathioprine, oral corticosteroids, systemic retinoids, psoralen plus ultraviolet (UV), or UVB phototherapy. The non-biologic/non-MTX cohort includes patients who are receiving other systemic immunomodulators (including cyclosporine, tacrolimus, mycophenolate mofetil, other immunomodulators, and oral corticosteroids) at entry or start other immunomodulators after registry and who haven't been exposed to MTX previously or concurrently; patients who receive only topical and/or phototherapy at/after registry entry are also in this cohort.  ^d^ Includes “Other biologics” group (n=54); data not shown.  ^e^ Data were collected at baseline (defined as the last non-missing value that is closest and prior to/on the cohort start date).  ^f^ History of infections is defined as infections within 3 years of enrollment and required a prescription medication. Obesity class based upon National Heart Lung and Blood Institute Obesity Education Initiative - <http://www.nhlbi.nih.gov/health/public/heart/obesity/lose_wt/bmi_dis.html> | | | | | | | | |
